# Critical Test of the Beneficial Consequences of Lifting the Ban on Direct-to-Consumer Advertising for Prescription Drugs in Italy: Experimental Exposure and Questionnaire Study

**DOI:** 10.2196/40616

**Published:** 2023-07-17

**Authors:** Peter Johannes Schulz, Francesca Crosignani, Serena Petrocchi

**Affiliations:** 1 Faculty of Communication, Culture and Society Università della Svizzera italiana Lugano Switzerland; 2 Department of Communication & Media Ewha Womans University Seoul Republic of Korea; 3 Wee Kim Wee School of Communication and Information & Lee Kong Chian School of Medicine Nanyang Technological University Singapore Singapore; 4 Faculty of Biomedical Sciences Università della Svizzera italiana Lugano Switzerland

**Keywords:** eDTCA, health literacy, knowledge, empowerment, health information, antidepressant, depression, depressive disorder, pharmaceutical, advertise, advertising, drug, marketing, patient education, consumer, health education

## Abstract

**Background:**

There are only two countries in the world (the United States and New Zealand) that allow the pharmaceutical branch to advertise prescription medication directly to consumers. There is pressure on governments to allow direct-to-consumer advertising (DTCA) for prescription drugs elsewhere too. One argument the industry uses frequently is the claim that exposure to DCTA, through various methods and occasions, is supposed to improve customers’ knowledge of a disease and treatment. This argument has been part of the health care community’s wider discussion of whether DTCA of prescription drugs benefits the population’s general interest or is only an attempt to increase the sales of the pharmaceutical branch. Belief in true learning by DTCA is rooted in concepts of empowered consumers and their autonomous and empowered decision-making.

**Objective:**

In this study, we tested the hypotheses that contact with DTCA increases recipients’ literacy/knowledge, especially regarding the side effects of treatment (hypothesis 1), and empowerment (hypothesis 2). We further hypothesized that DTCA exposure would not increase depression knowledge (ie, about treatments, symptoms, and prevalence) (hypothesis 3).

**Methods:**

A snowball sample of 180 participants was randomly split into three experimental groups receiving (1) a traditional information sheet, (2) a DTCA video clip for an antidepressant prescription drug, or (3) both. The video was original material from the United States translated into Italian for the experiment. Dependent variables were measures of depression knowledge (regarding treatments, symptoms and prevalence, and antidepressant side effects), depression literacy, and empowerment.

**Results:**

None of the experimental groups differed significantly from the others in the empowerment measure (hypothesis 2 not confirmed). Partial confirmation of hypothesis 1 was obtained. Lower values on the depression literacy scale were obtained when participants had been given the video compared to the sheet condition. However, the general depression knowledge and its subscale on side effects reached higher scores when participants were exposed to the DTCA, alone or in combination with the information sheet. Finally, participants showed lower scores on knowledge about treatment and symptoms or prevalence after watching the video compared to the sheet condition (hypothesis 3 confirmed). Symptoms and prevalence knowledge increased only when the video was presented in combination with the sheet.

**Conclusions:**

There is no evidence for an increase in empowerment following DTCA exposure. An increase in knowledge of the side effects of the medication was observed in the group exposed to the DTCA video. This was the only result that confirmed the hypothesis of the beneficial effect of DTCA videos on knowledge. Written information proved to be the most suitable way to convey knowledge on treatments and symptoms prevalence. Our findings support the necessity of studying health literacy and patient empowerment together and the consequences of such an increase in knowledge in terms of help-seeking behavior.

## Introduction

### Background

Pharmacological companies, especially those in the United States, spend a lot of money on direct-to-consumer advertising (DTCA) of prescription drugs. DTCA is typically described as any promotional attempt by pharmaceutical companies to expose the public to prescription drug information using media. Advertisements and videos appear in newspapers, magazines, television, radio, video, internet websites, nonmedical journals, pharmacy brochures, and directly mailed letters. A frequently used typology is based on criteria such as a central message, the naming of the brand, and situational factors. The advantages ascribed to DTCA were reported to include cognitive (to inform), affective (to persuade), and behavioral (to remind consumers to take action) aspects [1].

All other countries except for New Zealand and, to a lesser extent, Canada [2] ban this form of advertising; however, companies and their representatives have run campaigns to lift the ban. The ban has been upheld everywhere, most importantly in the European Union 15 years ago when 22 of the 27 member nations voted against liberalization [3-5]. For the past two decades, the question was raised whether the ban on DTCA for prescription drugs served the public’s well-being or missed a chance to inform people. In short, the question was whether DTCA for prescription drugs was primarily intended to provide unbiased information to consumers or whether its actual purpose was to raise revenue for pharmaceutical companies. However, this was far from being the end of the controversy [6,7] since several changes affected all areas of communication through the immense progress of digital communication and digitalization.

A first consideration is the basic observation that digital communication cannot be stopped at national borders [8]. A government agency with competence for regulation of advertising pharmacological products can prohibit DTCA, but it cannot realistically prevent DTCA from being watched or listened to in their country by their citizens. Online marketing allows the pharmaceutical industry to reach a larger number of consumers than traditional forms of communication (ie, television, newspapers, or via their doctors) [8] and the budget dedicated to promoting drugs online has increased over time [9]. A second aspect is the development of interactive programs and websites (ie, so-called eDTCA and eDTCA 2.0). Online forms of drug advertising can be made fully available via social networks and are globally accessible [8]. Presenting drug information via eDTCA in a fair and balanced way is a challenge and a major problem for public health. Studies have revealed that websites and online marketing often do not present all available risk information about the drugs or at least present these risks in an unbalanced way compared to the benefits [1,10]. A content analysis of posts on social media also found a high presence of medication issues; when DTCA on drugs were found, the majority of them did not follow fair-balance rules [11]. Moreover, companies hiding their affiliations and controlling users’ comments in online advertising are established practices with detrimental effects on how people evaluate and spread drug information [12]. This may pose a high health risk for a significant portion of the population [13].

The aim of this study was to address the question of how the European public might react were the ban on DTCA for prescription drugs to be lifted.

### Earlier Research on the Effects of DTCA

Prior research demonstrated that DTCA increases medication sales in the United States, with a risk of potential overuse of drugs [14]. The US General Accounting Office found 8.5 million prescriptions every year for each advertised drug [15]. Mintzes et al [16] compared prescription decisions in a US setting where DTCA was legal with those in a Canadian setting where it was not. They found that more advertising leads to more requests for the advertised medicines and to more prescriptions. Indeed, if patients, after watching a prescription drug advertisement, go to the doctor and ask for this very medication to be prescribed, it is easy to imagine that the visit would end with a prescription signed [17]. Moreover, a systematic review led to the conclusion that advertising affects patients’ information-seeking and request for specific drugs and doctors’ availability in prescribing [18]. A survey study compared doctors’, pharmacists’, and patients’ perceptions and attitudes on DTCA in New Zealand, a country that allows DTCA of prescription medication, with those of a sample Belgium that does not allow DTCA [17]. There was more criticism than support in both countries, but significantly more support in New Zealand than in Belgium. DTCA did not substantially affect the patients’ behavior or their interaction with doctors and pharmacists in either country. Patients were aware of shortcomings in DTCA, as are doctors and pharmacists. Particularly, participants were not convinced of the information function DTCA could serve and further felt that DTCA was not a reliable source of information. However, shortcomings did not reach as far as doing harm to the self-perceived relationship between doctors, pharmacists, and patients. As could be expected, compared with their Belgian colleagues, New Zealand physicians were more often confronted with patients’ inquiries or demands concerning prescription drugs. They also prescribed drugs more often [19]. In another study, Diehl et al [20] confirmed that US participants showed more positive attitudes and less skepticism toward DTCA compared to those of German participants who are not exposed to drug advertisements.

The principle issue of DTCA is about its persuasive impact on consumers, and about the fact that certain advertisements create impressions of effectiveness that are misleading and report important safety information in an incorrect way. Rubinelli et al [19] found that DTCA presents information that is framed in persuasive and argumentative structures that are potentially misleading. Drug advertising presents standpoints, which are usually variants of “Ask your doctor about medication X.” Several studies highlight the importance of the ability to recognize advertising for what it is [14,19,21]. The impressive development of online forms of DTCA may determine an exacerbation of the negative effects of the more traditional forms of DCTA, as already discussed. eDTCA is directly and fully available even in countries in which DTCA is forbidden, thereby exposing the public to some possible negative effects. The habit to overemphasize the benefits of a drug and hide the downsides is one main risk for individuals [22] with the consequence that patients do not have the right information to evaluate the risks for their health. Indeed, risk information is often not clearly visible and visitors need to go to the end of the page to see this information, while benefits information is presented in a more appealing and eye-catching way [23,24]. Even when the information is given, there is no guarantee that patients will be able to understand it or to distinguish between reliable and unreliable information. Another important point is that this form of marketing provides people with the tendency to present complex information in a simplified way [6], which is especially true for controversial products [25]. Real-world experience with depression also affects the perception of DTCA. In a survey study of college students, An et al [26] found that among participants who had no experience of depression, high exposure to antidepressant videos on television improved respondents’ ability to recognize depression cases, increased their awareness of treatment with antidepressants, and augmented their evaluation of this type of drug. Park et al [27] found that DTCA could increase the perception to be at risk of developing clinical depression in the future, especially when participants are less skeptical of prescription drug advertising.

### Health Literacy and Empowerment

Health literacy may be considered the most important predictor and component of the social determinants of health. The World Health Organization stated that health literacy is “a stronger predictor of an individual’s health status than income, employment status, education, and racial or ethnic group” [28]. The term health literacy was proposed by Simonds [28] and its definition was updated in 2020 in the Healthy People 2030 documentation [29], in which the concept was divided into personal health literacy and organizational health literacy. The former is defined by Healthy People 2030 as the degree to which individuals have the ability to find, understand, and use information and services to inform health-related decisions and actions for themselves and others, while the latter is defined as the degree to which organizations equitably enable individuals to find, understand, and use information and services to inform health-related decisions. These new definitions of health literacy highlight the importance of not simply *understanding* but also properly *using* health information. In this paper, we mostly consider the personal dimension when referring to health literacy.

Health literacy is generally considered a set of knowledge, skills, or a hierarchy of functions; most of the studies in this field to date have focused on basic health literacy and numeracy skills, as components of so-called functional literacy. Indeed, the tests most commonly used to measure health literacy, the Test of Functional Health Literacy in Adults [30] and Rapid Estimate of Adult Literacy in Medicine [31], use these specific abilities.

Patient knowledge is nowadays often considered a part of the wider concept of health literacy. The situational conditions of the controversy over DTCA for prescription drugs lead, as we have seen, to claims that lifting the ban would result in people becoming better informed about medical subjects. It can certainly be assumed that medical decisions are based on knowledge, almost exclusively the health care provider’s knowledge until recently and increasingly the patients’ knowledge as patients become more active in participating in medical decision-making [32]. Knowledge can be considered the key concept of health literacy. Schulz and Nakamoto [33] identify health literacy as a set of basic literacy, procedural knowledge, declarative knowledge, and judgment skills [33].

Our research interest is to find out whether a lift of the ban on DTCA for prescription drugs increases health literacy (to which knowledge belongs). A systematic review provided substantial evidence for DTCA prompting information-seeking in patients [34]. This could be interpreted as indicating a failure of advertising to convey information to patients, who then have to obtain the information on their own. More realistically, this could be interpreted as elucidating the process that generates the acquisition of knowledge, as advocated by supporters of DTCA, who argue that it is a response to advertisements and videos targeted toward consumers. The argument that health care consumers will profit in the long run from exposure to DTCA because exposure will extend their knowledge might be undermined by another, more obligatory argument. This reflects the assertion that consumers, or at least some of them, will command health literacy at a level that allows participation in health decisions. Put differently, directing advertising at health care consumers who show very low levels of health literacy that prevent them from taking part in health decisions in a meaningful way should be avoided.

Throughout its existence as a scholarly construct, health literacy had a sister concept in (patient) empowerment [35]. Indeed, the concept of empowerment was considered together with health literacy. The term empowerment was described by Paulo Freire [36], which can be defined as the dynamic process through which individuals attain mastery and exercise control over their own lives [35]. In particular, patient empowerment was defined by the World Health Organization in 1998 as “a process through which people gain greater control over decisions and actions affecting their health” [35]. Psychological empowerment is an important aspect of this concept, referring to the subjective feelings of empowerment wherein people think about their self-perception and perceived competence in effectively executing tasks, understanding situations, and feeling themselves able to make autonomous decisions.

If empowerment used to be understood as something that was very much like health literacy, something that need not be differentiated, and something the relationship of which did not need any intellectual effort, the relationship between health literacy and empowerment could well be left alone. However, the view seems to be gaining ground that the two constructs will not necessarily be in a harmonious situation. This is especially so when individuals show high empowerment coupled with low health literacy [33]. Individuals in this category will be tempted to try out the reach of their decision-making without possessing a basis for selecting a decision that does not harm them. Schulz and Nakamoto [33] named this class of health care consumers “dangerous self-managers.” People with high empowerment but low health literacy seem to report poor health status compared to literate individuals with low empowerment [37]. The evidence of this study [38] suggested the independence between health literacy and empowerment.

### General Hypotheses and Research Questions

This study contributes to the real-world problem of the ban on DTCA for prescription drugs being lifted. The novelty of the study consists in differentiating the type of effects generated by DTCA, because it has been asked whether DTCA could have a differential consequence according to the type of knowledge considered. As the health topic for studying the questions raised here, we selected depression and antidepressants because this is a highly sensitive sector.

The hypotheses are based on the claim that contact with these ads, foremost the videos, will increase recipients’ knowledge of the (depression) condition, especially regarding the side effects of treatment (hypothesis 1) and empowerment (hypothesis 2). However, the video was not expected to increase more general knowledge (ie, about treatments, symptoms, and prevalence) (hypothesis 3).

## Methods

### Experimental Design

This experimental study aimed to test the impact on health literacy and empowerment of a DTCA video for a prescription medication used to treat depression. The experimental factor in the main study was manipulated and participants were randomized into three different conditions (DTCA video vs text vs DTCA video+text). This represents an incomplete full factorial design (ie, a 2 [absence/presence]×2 [video/information sheet] design) with the no video/no information sheet group missing. The incomplete structure was chosen as the missing group would represent people not exposed to any kind of information (neither via reading material nor video), and such people hardly exist. This choice was based on the almost complete absence of such a design in relevant studies [38]. The study was conducted in Italy, a country in which DTCA is banned. This provides the perfect setting to test whether knowledge and empowerment are positively influenced by drug advertising.

### Ethical Approval

The questionnaire included an initial introductory page describing the context and the aims of the research. Participants were also informed on the respect of the right to privacy, specifying that all data collected will be kept anonymous and used exclusively for the research. Before starting with the questionnaire, they granted their participation via the online form. In compliance with the ethical rules defined in the Declaration of Helsinki, the possibility of interrupting the completion of the survey and withdrawing from the research at any time was guaranteed and made explicit in writing. Moreover, in case of further doubt and/or questions, authors’ contacts were provided, making it possible to satisfy every need of the participants. The data were collected anonymously and participants did not receive any compensation for their participation. The local Ethical Committee of the Università della Svizzera Italiana approved the study (CE_2022_15).

### Experimental Material

The DTCA was an Italian translation, with subtitles, of a US advertising video for Rexulti, a real drug for treating depression that is essentially unknown in Italy. This video was chosen according to an accurate analysis of different factors. First, it is a real video and not a mock solution, which further considers a drug for depression that is unknown in Italy. Second, the Rexulti video stresses empowerment and partly literacy. Moreover, the video is clear, with a good graphic design, easy to understand to everyone, and not too long or too short. In the video, the main character is a depressed woman in her 40s. She talks about her depression and explains that she had taken an antidepressant before for months but she was still depressed; therefore, she went to her doctor who suggested that she add Rexulti to her antidepressants. The side effects of Rexulti are then explained with an accelerated speed. The link to the video is provided in Multimedia Appendix 1 (Description of the Experimental Manipulation).

The information sheet (see Multimedia Appendix 1, Description of the Experimental Manipulation) provided a brief description of the most important characteristics of depression, its major symptoms, and possible treatments. The text was written in the Italian language and the information was taken from the website of the World Health Organization in its section about mental health [39].

### Sample

To detect an effect size of 0.38 with 95% power (α=.05), G*Power suggested a total sample size of 173. The effect size was calculated according to a previous study on 80 healthy adults examining the effect of empowerment and health literacy on health status [38]. The goal was to reach approximately 180 participants with approximately 60 for each condition. A total of 274 people participated in the survey; 94 responses were not completed and were excluded from the analysis. Therefore, a total of 180 responses were analyzed, with 55 in the DTCA condition, 67 in the information sheet condition, and 57 in the condition of receiving both. Participants were adults ranging in age from 18 to 83 years (mean 39.47, SD 16.34 years). Since the pilot study was not published, we also provide a posthoc analysis based on α=.05, N=180, 3 groups, the means of the treatment knowledge variable (as the most conservative ones), and a pooled SD of 0.38. This led to an estimated effect size of 0.25, which is more conservative compared to the effect size of the a priori power analysis, but still to a sample size of 177. This result would give us the confidence that the evidence from this study could be trusted.

### Procedures

An online survey was developed on Qualtrics, a secure online software that allows one to create questionnaires and then share them with participants through a link on social networks (eg, Facebook and WhatsApp). The link was spread to personal contacts, to Facebook groups, and on the Facebook profile of the research group owing to sponsored advertising. The questionnaire was published on November 15, 2021, and remained open for 20 days, until December 5, 2021. Qualtrics has a randomizer tool that allowed participants to be randomly assigned to one of the three conditions (DTCA video vs text vs DTCA video+text) immediately after having acknowledged participating in the study. There were no missing data because participants were forced to answer each question before going on to the subsequent question.

Immediately after the manipulation, we ran one manipulation check for the text and one for the video, using two questions each. When the condition included both the video and the text, the manipulation check included all the questions presented below. For the video, one question asked the participant to choose among three possible answers to best describe what they had learned (ie, “The woman has never taken an antidepressant before even though she has been depressed for months”; “The woman has already taken an antidepressant, but she still feels depressed”; “The woman has already taken an antidepressant that has helped to cure the depression”). The second question asked the participant to choose the correct option among the following possibilities: “Rexulti is a drug that does not need a medical prescription,” “Antidepressants do not have any contraindications for subjects aged less than 25 years,” “Rexulti increases death and ictus risk in older patients.” The two questions for the text asked if depression has “just physical symptoms,” “just mental symptoms,” “both physical and mental symptoms,” and if the most common symptom of depression is “negative thoughts,” “tremors,” or “nausea.” A total of two questionnaires were discarded after an unsatisfactory check result.

After the manipulation check, the participants answered items on depression knowledge, depression literacy, and empowerment. At the end of the survey, we thanked participants for their time and availability and we debriefed them. Three links with detailed information about depression were provided for participants interested in the topic. We also explained to participants who had seen the video that it was just an example used for the research and that the information may not be true in Italy. Moreover, we specified that it was not possible to buy antidepressants in Italy without a medical prescription and we suggested in case of depressive symptoms to contact a doctor. Authors’ emails were provided in case of doubts or questions.

Before the questionnaire went to the field, a brief pilot test was run to collect qualitative data on the intelligibility of words used in the questions, clarity, and understandability of the questions. The pilot test was conducted on six people and revealed that the terms used in formulating the questions were unambiguous and that the questions were formulated in a clear and understandable way.

A pretest was performed to assess if the materials of the manipulation were easily comprehensible to everyone and clear. Both the video and the text were sent to four people of different ages and educational levels (woman, 80 years old with a low educational level; woman, 50 years old; man, 20 years old who was a university graduate; and man, 50 years old). The participants judged the material as clear and understandable.

### Measures

For the measure of *depression knowledge*, participants were asked to answer eight questions created ad hoc for this research. The first four items concerned general knowledge about how depression manifests itself, symptoms, treatments, and basic data on the prevalence (eg, “Depression only affects the emotional sphere and the mood of the depressed person,” “Depression can only be treated by a pharmacological approach,” “Depression affects women more than men”). The other four questions were drafted starting from information presented in the Rexulti video but rephrased to make them as general as possible so that even participants who had not seen the video could answer (eg, “Antidepressants can cause side effects, but they are not serious”; “Antidepressant drugs can worsen depression in people under the age of 25”). The indicator was the number of correctly answered questions among the eight. Cronbach α for this questionnaire was low (see Table 1). Therefore, a factor analysis was performed to examine the structure of the scale. Detailed results of the factor analysis are reported in Table S1 of Multimedia Appendix 1. Three factors were extracted, as reported in Table 1: one focusing on the specific knowledge regarding treatments, one about symptoms and prevalence, and the last on knowledge about antidepressant side effects.

**Table 1:** Dependent measures.

Measure		Cronbach α	Items, n	Possible score range	Score, mean (SD)	Measure scale	Interpretation of high values
**Depression knowledge**							
	Overall	.50	8	0-8	5.14 (0.11)	True/false/dk^a^; correct answers (n)	Good knowledge
	Treatments knowledge	.33	2	0-2	1.80 (0.04)	True/false/dk; correct answers (n)	Good knowledge
	Symptoms and prevalence knowledge	.49	2	0-2	1.48 (0.053)	True/false/dk; correct answers (n)	Good knowledge
	Side effects knowledge	.45	3	0-3	1.70 (0.08)	True/false/dk; correct answers (n)	Good knowledge
Depression literacy		.77	22	0-22	13.30 (3.9)	True/false/dk; correct answers (n)	High literacy
**Empowerment**							
	Overall	.75	10	10-40	11.32 (5.6)	5-point Likert scale	High confidence and autonomy
	Confidence in autonomous depression management	.86	4	4-16	5.25 (0.26)	5-point Likert scale	High confidence
	Confidence in ability to get an antidepressant	.82	3	3-12	4.19 (0.20)	5-point Likert scale	High confidence
	Put trust in specialists	.96	2	2-8	1.18 (1.64)	5-point Likert scale	High trust

^a^dk: Don’t know.

As a measure of *depression literacy*, participants were asked to fill out the Depression Literacy Questionnaire [40,41], a 22-item scale with the aim to assess mental health literacy specific to depression. Items were created to cover all general knowledge of depression; in particular, the scale aims to assess depression literacy based on symptomatology, establishing a difference between biological, cognitive, behavioral, and psychotic symptoms; impacts; and management of depression. In previous research [41], the Depression Literacy Questionnaire showed good internal consistency reliability (Cronbach α=.70) and good test-retest reliability calculated with the Pearson correlation coefficient (*r*=0.71, *P*=.02). The indicator was the number of correctly answered questions among the eight (see Table 1 for a description). Table 2 reports correlations between depression literacy and the depression knowledge scale, including its subscales.

**Table 2:** Correlations between the depression knowledge scale and subscales and depression literacy.

Variable			Depression knowledge (total)		Depression knowledge (treatments)		Depression knowledge (symptoms and prevalence)		Depression knowledge (side effects)		Depression literacy
**Depression knowledge (total)**											
	*r*	1		0.28		0.54		0.77		0.33	
	*P* value	—^a^		<.001		<.001		<.001		<.001	
**Depression knowledge (treatments)**											
	*r*	0.28		1		0.07		0.04		0.09	
	*P* value	<.001		—		.33		.56		.18	
**Depression knowledge (symptoms and prevalence)**											
	*r*	0.54		0.07		1		0.02		0.28	
	*P* value	<.001		.33		—		.81		<.001	
**Depression knowledge (side effects)**											
	*r*	0.77		0.04		0.02		1		0.18	
	*P* value	<.001		.54		.81		—		<.001	
**Depression literacy**											
	*r*	0.33		0.09		0.28		0.18		1	
	*P* value	<.001		.18		<.001		<.001		—	

^a^Not applicable.

*Empowerment* was a measure made up of 10 items, created ad hoc for this research, which aimed to assess the participants’ empowerment. The measure was created as an adaptation of other measures [33,42,43] evaluating the competence, meaning, impact, and self-determination dimensions of empowerment. For the specific purposes of this study, we focused on the competence dimension and decision autonomy. The 10 items were developed ad hoc for this research since the existing items do not apply to the specific context of this experiment (ie, the Rexulti video). Three dimensions of competence and decision autonomy were explored: (1) autonomy in managing depression (4 items), (2) autonomy in the ability to get an antidepressant (4 items), and (3) autonomy in decision-making and asking for help to a physician (2 items). Response options were on a 5-point Likert scale, ranging from 1 (“completely agree”) to 5 (“completely disagree”). To obtain a total score that describes the level of empowerment and decision autonomy across the three factors, the scores for each respondent were summed. Similarly, a measure of empowerment based on all items was obtained (see Table 1). A factor analysis was performed to examine the structure of the scale and demonstrate the three expected factors. Detailed results of the factor analysis are reported in Table S2 of Multimedia Appendix 1.

### Analyses

Descriptive statistics in the form of means, SDs, ranges, and α levels have been provided. A principal component analysis with varimax rotation was carried out on the items of the depression knowledge scale and the items of the empowerment scale (results are reported in Multimedia Appendix 1). Univariate ANOVA was performed to compare the dependent variables for the different manipulated conditions. These analyses were used to test hypotheses 1, 2, and 3. The means of each competence (information sheet only, DTCA exposure only, and the combination of both) were calculated and the comparisons between the means were carried out considering the sheet only as the referring condition. The posthoc Scheffe test was applied for these comparisons.

## Results

Participants were mostly female (132/180, 73.3%). Participants between 18 and 83 years of age participated in the study; the mean age was 39.47 (SD 16.34) years. Most of the respondents fell into the age slots of 18 to 29 years (n=84, 46.7%), followed by 50 to 59 years (n=52, 28.9%). A share of 58.3% (n=105) was employed, while 46.4% (n=84) of the sample had a partner. The majority of respondents held a bachelor’s or master’s degree (n=121, 69.4%) or a high school diploma (n=53, 29.2%). The majority of participants did not have any chronic illness (n=136, 76.0%), and most of them considered their health status as good or very good (n=102, 56.2%) or normal (n=68, 38%), whereas no participant identified their health status as very poor. Most respondents (n=120, 67.0%) had not seen their doctor in the last 6 months.

All five analyses we ran on respective communication capabilities seemed to show the effects of exposure to DTCA on knowledge and depression literacy. The depression knowledge in the DTCA-only condition was not significantly different from that of the sheet-only condition. However, the combination of the sheet and DTCA resulted in an increase in depression knowledge compared to that of the sheet-only condition. On the other side, depression literacy was lower in the DTCA exposure condition compared to that of the sheet-only condition. The four analyses run to track effects on empowerment did not show any significant effect considering both the full score and the three subscores. ANOVA results are provided in Table 3.

**Table 3:** Health communication competence as predicted by the type of medium.

Information condition		Sheet only, mean (SE)	DTCA^a^ only				Sheet and DTCA				ANOVA		
			Mean (SE)	ΔM^b^	*P* value^c^	Mean (SE)		ΔM	*P* value^c^	*F*_2,178_		*P* value^d^	
**Depression knowledge**													
	Total	4.74 (0.155)	4.82 (0.20)	0.08	.95	5.93 (0.13)		1.19	<.001	12.987		<.001	
	Treatment knowledge	1.91 (0.034)	1.67 (0.08)	–0.24	.02	1.79 (0.03)		–0.12	.32	4.272		.02	
	Symptom and prevalence knowledge	1.70 (0.07)	0.93 (0.10)	–0.77	<.001	1.75 (0.05)		0.06	<.001	32.342		<.001	
	Side effects knowledge	1.01 (0.12)	2.09 (0.13)	1.08	<.001	2.14 (0.08)		1.13	<.001	28.430		<.001	
Depression literacy		13.67 (0.49)	11.93 (0.54)	–1.74	.04	14.19 (0.29)		0.52	.74	5.463		.005	
**Empowerment**													
	Total	11.97 (0.07)	10.18 (0.07)	–1.79	.22	11.63 (0.08)		0.04	.95	1.702		.19	
	Confidence in autonomous depression management	5.57 (0.44)	4.80 (0.44)	–0.77	.49	5.39 (0.49)		–0.26	.93	0.723		.49	
	Confidence in ability to get an antidepressant	4.26 (0.31)	3.87 (0.34)	–0.39	.73	4.42 (0.38)		0.20	.95	0.620		.54	
	Put trust in specialists	1.36 (0.22)	1.02 (0.22)	–0.34	.51	1.11 (0.18)		–0.25	.68	0.747		.48	

^a^DTCA: direct-to-consumer advertising.

^b^ΔM: Mean difference from sheet-only condition.

^c^*P* value relates to the mean difference (ie, one column to the left) calculated with the posthoc Scheffe test.

^d^*P* value relates to the communication competences in the first column as the dependent variable and the experimental conditions as the independent variable.

These general results would suggest mixed support for hypothesis 1 and contradiction to hypothesis 2. In brief, there is evidence for an increase in depression knowledge, but not depression literacy, after being exposed to a DTCA video while empowerment remains stable. However, the actual findings are more nuanced.

Participants showed lower scores in the treatment knowledge and symptoms and prevalence knowledge subscales when they were given the DTCA video instead of the classic information sheet, and the low scores did not recover completely when both the sheet and video were offered. Side-effects knowledge was high when the video was presented, alone or together with the traditional sheet, and it was low when no DTCA video was offered (Table 3).

## Discussion

Among the arguments frequently used by advocates of DTCA for prescription drugs, there is the claim that the devices used for this type of advertising, videos foremost, will increase consumers’ knowledge and empowerment. We tested these assumptions in a survey experiment and did not find evidence to support them.

This study found mixed evidence for hypotheses 1 and 3. We found that the combination between written information and exposure to a DTCA video increases the general level of knowledge of individuals, but also that participants exposed to the DTCA-only condition showed lower depression literacy compared to that of participants exposed to the sheet-only condition. Moreover, when considering the various subdimensions of knowledge, the exposure to the video increased only the ability to correctly identify the knowledge of side effects. When knowledge about treatments and symptoms prevalence were considered, we found that written information resulted in a greater increase in individuals’ knowledge than the exposure to the DTCA video.

When empowerment was considered, the three conditions of the experimental design did not influence any of the subdimensions (ie, autonomy in managing depression, autonomy in the ability to get an antidepressant, and autonomy in decision-making and asking for help to a physician). This means that hypothesis 2 could not be confirmed.

The present experimental study took, as its starting point, the claim that health care consumers gain medical knowledge of their condition and, importantly, its treatment via exposure to commercials about drugs. Our study did not produce substantial evidence in favor of the claim. The capacity of DTCA videos to improve knowledge, via a single exposure, does not emerge as in any way superior to a single exposure to a traditional mode of conveying information. A single exposure to a DTCA is therefore not an effective vehicle for increasing the knowledge of consumers; rather, the combination of information presented in a video and in a text is the best solution to increase people’s knowledge. Compared to other studies in the literature [26,27], our study focused attention on several subdimensions of knowledge and replicated the previous findings when considering the general knowledge, but found different patterns when more specific subdimensions are considered. This suggests that written information should be preferred when one wants to be informed about treatment options and symptoms prevalence.

The second general result is that, in contrast to the variety of effects in depression literacy, there were no effects at all in the empowerment measure. This might be a consequence of the measure employed. Some of the items in the measure formulated self-perceptions that are very much unlike anything a depressed person might feel (eg, feeling ready to face depression or feeling confident to manage depression). Other items were worded as confirmation of the respondents’ decision to ask for help to a physician. Seeking professional help is an ambiguous behavior in relation to the concept of empowerment. It can describe a case of a person who has to overcome resistance before seeing a specialist but it can also indicate insight into the limits of one’s capacity to help oneself.

This study has some limitations. First, all the measures were self-reported, and especially the empowerment scale has received little evidence of validity. Second, the experiment was conducted in Italy; therefore, the results cannot be generalized to other countries at this time and future research will be needed to extend the results to other geographical areas. Third, we assumed that baseline values of knowledge and empowerment were equal across groups due to randomization; however, without baseline measures of these variables, the claim could be fallacious. Fourth, although the content of the DTCA and the information sheet were comparable, some differences in the information presented in the commercial versus the information sheet may have influenced the results on the knowledge subscales. Another limitation includes the fact that we measured whether a *one-time* exposure to an existent commercial improves knowledge and empowerment. In real life, people are exposed to DTCAs many times a week or even a day. This could make a difference in individuals’ knowledge and empowerment compared to our experimental condition.

Our study provides evidence that traditional written information conveys knowledge on treatments for depression, symptoms, and prevalence better than DTCA videos. The separate measure of depression literacy also declined considerably after exposure to a DTCA video. The positive effect of DTCA on the side-effects knowledge may be triggered by a special focus on side-effects information included in the video. Finally, no effects were found on empowerment.

This study can serve as a starting point to create an effective and efficient strategy based on the development of health materials. The European Union voted against the liberalization of DTCA in the member countries. One can argue that lifting this ban on DTCA for prescription drugs would determine a positive influence on patients’ treatment decision-making in searching for help. However, lifting the ban would mean weakening the fundament of such decisions, since our study shows that the claimed compensation for such a weakening, increased depression literacy, will not materialize. Nevertheless, the balance remains negative.

## Appendix

Multimedia Appendix 1 Description of the experimental manipulation. Factor analyses on the depression knowledge scale and the empowerment scale.

Multimedia Appendix 2 Data set.

## Abbreviations

DTCA: direct-to-consumer advertising
eDTCA: electronic DTCA delivered via interactive programs and websites

## Data Availability

All data generated and/or analyzed in this study are available in the article and Multimedia Appendix 2.
