# Supplementary material for: Critical Test of the Beneficial Consequences of Lifting the Ban on Direct-to-Consumer Advertising for Prescription Drugs in Italy: Experimental Exposure and Questionnaire Study
Source: J Med Internet Res. 2023 Jul 17;25:e40616. doi: 10.2196/40616 (PMC10390971; doi:10.2196/40616)
Supplement: Multimedia Appendix 1 [file jmir_v25i1e40616_app1.docx]

**Multimedia Appendix 1**

**Description of the experimental manipulation**

Text

Depression is a very common psychiatric disorder, with more than 264 million people of all ages suffering from it worldwide. Depression affects not only the individual’s mood and emotional sphere but also the body. Common manifestations are physical symptoms and behaviors. Depression affects women the most. There are different forms of depression, each of them with different characteristics. The symptoms vary both according to the depressive form that affects the patient and the severity of the disorder itself. The most common symptoms of depression are frustration, insomnia, depressed mood, marked daily sadness, negative thoughts, loss of pleasure, and interest in any activity. The treatment of depression depends on many different factors, such as the depressive form affecting the patient and the severity. In general, depressive disorders can be effectively treated through a psychotherapeutic and/or pharmacological approach.

Video

Please see the link of the video stored on youtube (as a private video): https://youtu.be/Rvr3Id8eWtY

**Factor analysis**

Results of the factor analysis were carried out on the depression knowledge scale and empowerment scale.

**Factor analysis on the depression knowledge scale**

The depression knowledge scale was subjected to a principal component analysis with a varimax rotation. The Kaiser-Meyer-Olkin measure (KMO), verifying the sampling adequacy for the analysis, was 0.60, which is within the acceptable limit [1]. Three factors were extracted with eigenvalues above Kaiser’s criterion of 1. Table S1 showed the results of the analysis. The model explains 54.89% of the variance. Item clustering suggested that factor 1 (item 1 and item 2) represented the knowledge of depression treatments and what depression involves(24.38% of the variance), factor 2 (item 3 and item 4) represents knowledge of depression symptoms and prevalence in women (16.16% of the variance), factor 3 (item 6, item 7 and item 8) represents the knowledge about antidepressant side effects (14.34% of the variance). Item 5 was deleted because it had factor loadings higher than .30 for all three factors.

Table S1. Factor analyses on the items of the knowledge scale

| Knowledge scale | Factor 1  Treatments knowledge | Factor 2  Symptoms and prevalence knowledge | Factor3  Side effects knowledge |
| --- | --- | --- | --- |
| Depression only affects the emotional sphere and the mood of the depressed person. | .81 |  |  |
| Depression can only be treated by a pharmacological approach | .67 |  |  |
| Insomnia is one of the most common depressive symptoms. |  | .69 |  |
| Depression affects women more than men |  | .70 |  |
| Sometimes it is recommended to take two different antidepressants together to increase their effectiveness; in fact, depressive symptoms can be reduced by over 60% compared to taking a single antidepressant | .33 | .41 | .42 |
| Antidepressants can cause side effects, but they are not serious |  |  | .74 |
| Antidepressant drugs can worsen depression in people under the age of 25 |  |  | .81 |
| Antidepressant drugs can cause weight gain and heightened cholesterol levels |  |  | .41 |

**Factor Analysis on the Empowerment Scale**

The empowerment scale was subjected to a principal component analysis with a varimax rotation. The Kaiser-Meyer-Olkin measure (KMO) was 0.730 (see Table S2). Three factors have eigenvalues over Kaiser’s criterion of 1 and in combination explained 66.18% of the variance. Results show three factors (Table S2), Item clustering suggests that factor 1 (item 1, item 2, item 3, item 4) represents autonomy to manage depression (28.48% of the variance explained), factor 2 (item 5, item 6, item 7) represents autonomy in the decision-making about taking a drug to cure depression (21.05% of the variance explained), factor 3 (item 9 and item 10) represents the autonomy in decision-making about asking a physician for help in managing depression (16.65% of the variance explained). Item 8 was eliminated because it had factor loadings higher than .30 for two factors.

Table S2. Factor analysis on the items of the empowerment scale.

| Empowerment scale | Factor 1  Confidence in autonomous depression management | Factor 2  Confidence in ability to get an anti-depressant | Factor 3  Put trust in specialists |
| --- | --- | --- | --- |
| I would be able to handle difficulties and problems related to depression autonomously | .853 |  |  |
| I would be ready and able to face depression | .792 |  |  |
| I would have the required abilities to handle depression situations autonomously in the best way | .855 |  |  |
| I would be confident to manage depression and its difficulties autonomously. | .856 |  |  |
| I would insistently ask my doctor to prescribe an antidepressant |  | .830 |  |
| If my doctor refuses to prescribe an antidepressant, I will ask another physician |  | .864 |  |
| I think that an antidepressant would be the best solution to cure my depression |  | .801 |  |
| If it were allowed, I would directly go to a pharmacy to buy an antidepressant even if my doctor did not agree |  | .707 | .419 |
| I would entrust to a specialist to face depression |  |  | .963 |
| I would go to a specialist to handle depression in the best way |  |  | .960 |

**Reference**

1. Kaiser HF. A second generation little jiffy. Psychometrika 1970;35(4):401-415.
